# Trace Ru‐Doped PtCuRu@PtRu Core‐Shell Electrocatalyst for CO‐Resilient Methanol Oxidation

**DOI:** 10.1002/advs.75638

**Published:** 2026-05-15

**Authors:** Tianrui Xue, Shiyue Xing, Zhongliang Liu, Yiting Song, Jianyi Zhang, Miaomiao Liu, Huihui Li, Chunzhong Li

**Affiliations:** ^1^ Key Laboratory for Ultrafine Materials of Ministry of Education School of Chemical Engineering East China University of Science and Technology Shanghai P. R. China; ^2^ Shanghai Engineering Research Center of Hierarchical Nanomaterials School of Materials Science and Engineering East China University of Science and Technology Shanghai P. R. China; ^3^ Key Laboratory of Interfacial Physics and Technology Shanghai Institute of Applied Physics Chinese Academy of Sciences Shanghai P. R. China

**Keywords:** anti‐CO poisoning, bifunctional mechanism, core‐shell structure, methanol oxidation reaction

## Abstract

To overcome the limited water dissociation capability and transition metal dissolution in Pt‐based binary alloys during methanol oxidation reaction (MOR), a PtCuRu‐0.05@PtRu core‐shell electrocatalyst with trace Ru doping was synthesized via liquid‐phase reduction followed by *in‐situ* electrochemical dealloying. The design leverages three synergistic mechanisms: oxophilic Ru sites in the Pt‐rich shell facilitate water dissociation to generate *OH for efficient *CO oxidation; lattice mismatch between the ternary PtCuRu core and trace Ru‐doped Pt‐rich shell induces compressive strain, downshifting the Pt *d*‐band center to weaken *CO adsorption; and the Pt‐rich shell acts as a diffusion barrier suppressing Cu dissolution. As a result, PtCuRu‐0.05@PtRu delivers the highest mass activity of 1.208 A mg_Pt_^−1^, surpassing PtCu@Pt and commercial Pt/C samples. Moreover, PtCuRu‐0.05@PtRu exhibits superior durability, maintaining the highest current density during 3600 s chronoamperometry and showing only 3.31% activity decay after five consecutive stability tests (18,000 s), with preserved structural integrity. This work provides a viable strategy for simultaneously enhancing MOR activity and durability via synergistic composition and structure engineering.

## Graphical abstract

We engineered a trace Ru‐doped PtCuRu@PtRu core‐shell nanostructure. This design exploits lattice mismatch‐induced compressive strain between the PtCuRu ternary core and trace Ru‐doped Pt‐rich shell, systematically downshifting the Pt *d*‐band center to weaken *CO adsorption energetics. Concurrently, strategically incorporated oxophilic Ru sites facilitate spontaneous *OH generation at low potentials, enabling rapid oxidative removal of carbonaceous poisons through a bifunctional mechanism. This synergistic strain/ligand dual‐regulation approach collectively enhances MOR activity and CO tolerance while prolonging catalyst lifetime.

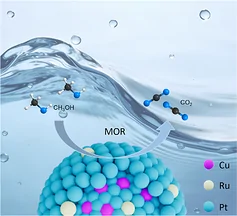

## 1 Introduction

Direct methanol fuel cells (DMFCs) are regarded as promising electrochemical power sources for portable electronic devices due to their high energy density, ease of fuel storage, and low operating temperatures [1, 2]. However, the commercial viability of DMFCs is fundamentally constrained by the sluggish kinetics of the anodic methanol oxidation reaction (MOR) [3, 4, 5]. While Platinum (Pt) is the most efficient catalyst for C─H bond cleavage, it suffers from severe CO poisoning, where *CO strongly adsorbs on Pt active sites, blocking further methanol adsorption and leading to rapid performance degradation [6, 7, 8, 9].

To mitigate CO poisoning, alloying Pt with 3d‐transition metals (TMs; e.g., Cu, Co, and Ni) has been widely explored [10, 11, 12, 13]. According to the *d*‐band center theory, incorporating TMs modulates the electronic structure of Pt via ligand effects, downshifting the Pt *d*‐band center relative to the Fermi level. This downshift weakens the Pt‐*CO binding strength, thereby promoting *CO desorption and restoring active sites. However, the CO tolerance cannot be fully rationalized by the electronic effect alone. Under the Langmuir‐Hinshelwood (L‐H) mechanism, the oxidative removal of *CO requires reaction with adjacent oxygenated intermediates (e.g., *OH) generated via water dissociation [4]. Many Pt‐TM binary alloys exhibit limited water dissociation kinetics at low potentials and thus insufficient *OH coverage, which kinetically hampers *CO oxidation and ultimately constrains MOR performance [6, 14]. Accordingly, introducing oxophilic components (e.g., Sn, Pb, and Ru) has been proposed to promote *OH formation at lower potentials and accelerate *CO stripping through a bifunctional mechanism [15, 16, 17]. Nevertheless, such non‐noble/oxophilic species are often thermodynamically unstable under acidic operating conditions, and their dissolution/leaching can compromise long‐term durability [18, 19].

To reconcile the activity‐durability trade‐off associated with the composition engineering in acidic media, constructing a core‐shell structure has emerged as an effective strategy [20, 21, 22]. A Pt‐rich shell can serve as a physical barrier that suppresses direct electrolyte attack on the underlying alloy/dopant species, thereby mitigating metal dissolution and improving durability. Meanwhile, lattice mismatch between a TM‐containing core (typically with a smaller lattice constant) and the Pt‐rich shell can introduce compressive strain, which further tunes the surface electronic structure of Pt and optimizes the adsorption strengths of key intermediates involved in MOR [23, 24].

Herein, we designed a trace Ru‐doped PtCuRu‐0.05@PtRu core‐shell electrocatalyst via liquid‐phase reduction followed by *in‐situ* electrochemical dealloying. This design synergistically integrates the strain effect and the bifunctional mechanism to optimize methanol oxidation performance. Specifically, the lattice mismatch between the ternary PtCuRu alloy core and the trace Ru‐doped Pt‐rich shell induces a persistent compressive strain, which downshifts the Pt *d*‐band center and weakens the adsorption of poisoning intermediates (e.g., *CO). Simultaneously, the oxophilic Ru sites within the Pt‐rich shell facilitate the generation of oxygenated species (*OH) at lower potentials, accelerating the oxidative removal of carbonaceous species. Consequently, the PtCuRu‐0.05@PtRu catalyst delivers a superior mass activity of 1.208 A mg_Pt_^−1^, representing a 1.4‐fold and 3.6‐fold enhancement over the Ru‐free PtCu@Pt and commercial Pt/C samples, respectively. Furthermore, the PtCuRu‐0.05@PtRu core‐shell electrocatalyst demonstrates exceptional long‐term durability and strong regenerability. After 18,000 s of cumulative operation, the mass activity decayed by only 3.31% with the structural and compositional integrity well‐preserved. These results underscore the critical role of coupling the compressive strain with trace Ru surface doping to achieve highly active and robust electrocatalysts for DMFCs.

## 2 Results and Discussion

### 2.1 Catalyst Synthesis and Characterization

**FIGURE 1:**
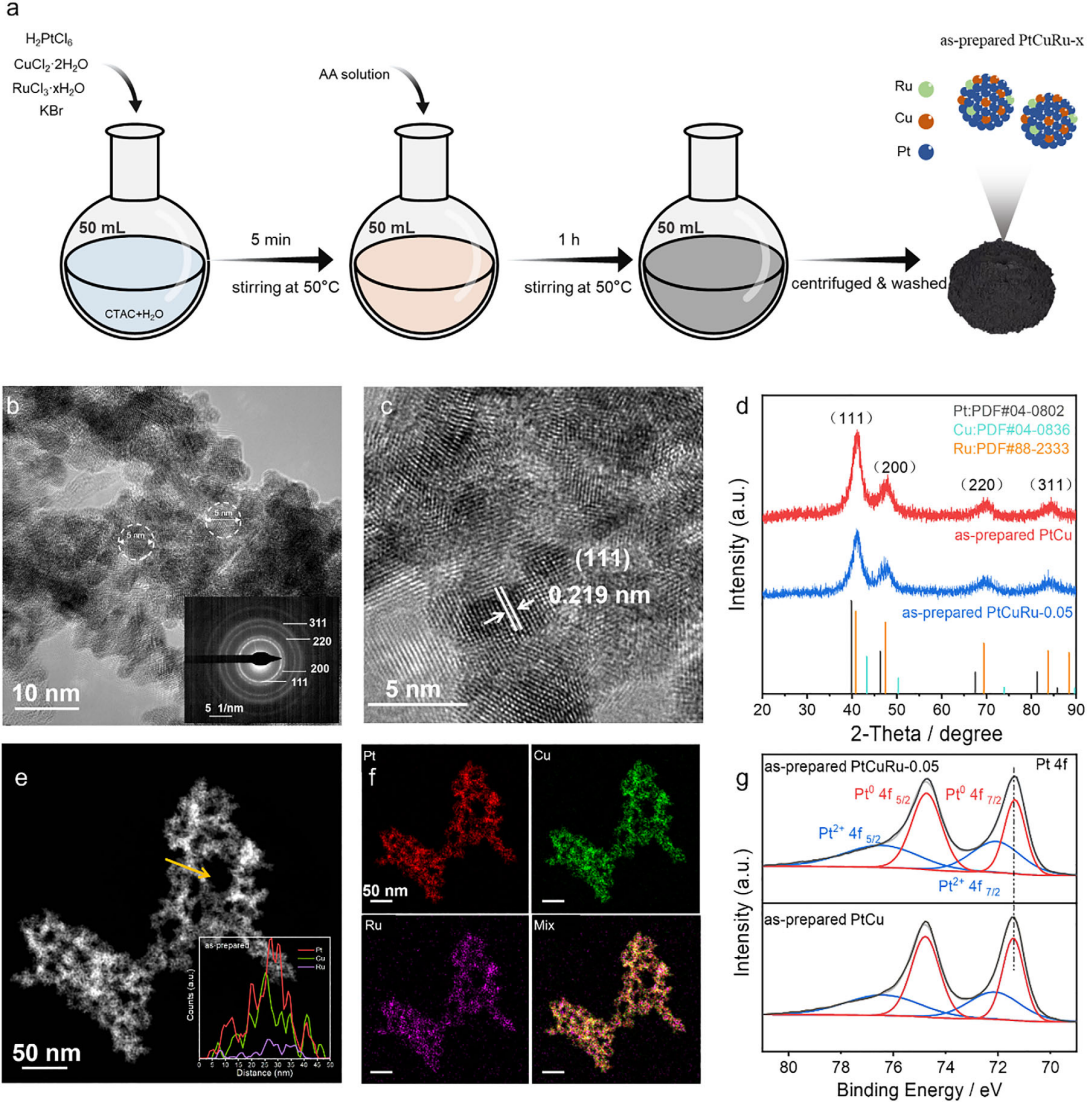
Characterization of as‐prepared PtCuRu‐x. (a) Schematic illustrating the synthesis of as‐prepared PtCuRu‐x alloy. (b) HRTEM image (inset shows the corresponding SAED pattern) and (c) lattice spacing analysis of as‐prepared PtCuRu‐0.05. (d) XRD patterns of as‐prepared PtCu and PtCuRu‐0.05. (e) HAADF‐STEM image (inset shows the corresponding EDS line‐scan analysis along the yellow arrow direction), and (f) EDS elemental mapping of as‐prepared PtCuRu‐0.05. (g) Pt 4*f* XPS spectra of as‐prepared PtCu and as‐prepared PtCuRu‐0.05.

The PtCuRu‐x@PtRu core‐shell electrocatalysts were synthesized via *in‐situ* electrochemical dealloying of the as‐prepared PtCuRu‐x alloy in 0.1 M HClO_4_. The as‐prepared PtCuRu‐x alloy was synthesized via a one‐pot liquid‐phase reduction method with a fixed Pt:Cu molar ratio of 1:1 (Figure 1a). The Ru stoichiometry (x) was achieved by adjusting the amount of RuCl_3_·xH_2_O precursors. The Ru‐free control sample (x = 0) of the as‐prepared PtCu is referred to as PtCu@Pt.

The morphology, elemental composition, and crystalline phase of the as‐prepared PtCuRu‐x alloy were first systematically characterized using transmission electron microscopy (TEM), high‐resolution TEM (HRTEM), X‐ray diffraction (XRD), and X‐ray photoelectron spectroscopy (XPS). The atomic ratios of Pt:Cu:Ru among all the as‐prepared catalysts were first determined using inductively coupled plasma‐optical emission spectroscopy (ICP‐OES) as shown in Table S1. The measured atomic ratios of Pt:Cu:Ru align closely with the precursors feeding ratios. While the Pt:Cu ratio maintains ∼1:1, the Ru content increases proportionally with elevated Ru precursor addition.

All the as‐prepared samples exhibit an interconnected branched‐chain architecture (Figure S1a–d) consisting of ultra‐small, cross‐linked grains. Based on the initial MOR performance screening, the as‐prepared PtCuRu‐0.05 alloy was identified as the optimal candidate and selected for further structural analysis (see Electrocatalytic performance measurements section). TEM imaging (Figure 1b) reveals that the as‐prepared PtCuRu‐0.05 is composed of randomly oriented nanoparticles with an average diameter of ∼5 nm. The polycrystalline nature of the framework is further confirmed by the selected‐area electron diffraction (SAED) pattern, which exhibits four well‐defined rings indexed to the (111), (200), (220), and (311) planes of a face‐centered cubic (fcc) lattice. Furthermore, HRTEM analysis (Figure 1c) displays clear lattice fringes with an interplanar spacing of 0.219 nm, corresponding to the (111) plane of the fcc lattice. This lattice contraction relative to fcc Pt(111) (0.226 nm) provides direct evidence for the successful incorporation of smaller Cu and Ru atoms into the Pt matrix to form a homogeneous alloy structure. Correspondingly, XRD patterns of as‐prepared PtCu and as‐prepared PtCuRu‐0.05 (Figure 1d) exhibit an fcc structure with diffraction peaks positioned between the reference patterns for fcc Pt, Cu, and Ru (JCPDS #04‐0802, #04‐0836, and #88‐2333). Both samples show noticeable peak shifts toward higher angles compared to fcc Pt, indicating lattice compression primarily induced by the incorporation of Cu atoms with a smaller atomic radius.

High‐angle annular dark‐field‐scanning transmission electron microscopy (HAADF‐STEM) and the corresponding energy‐dispersive X‐ray spectroscopy (EDS) mapping further confirm the uniform distribution of Pt, Cu, and Ru elements (Figure 1e,f) across the as‐prepared samples. EDS line‐scan profiles along the yellow arrow in Figure 1e reveal comparable signal intensities for Pt and Cu, whereas the Ru signal remains noticeably lower, consistent with its trace doping level. Quantitative EDS analysis indicates a Pt:Cu:Ru atomic ratio of 43.2:53.6:3.2, which is in good agreement with the bulk composition determined by ICP‐OES (45.1:53.6:1.2, Table S1). Atomic‐resolution HAADF‐STEM imaging (Figure S2a,c) resolves distinct elemental distributions through atomic‐number‐dependent intensity variations across lattice columns (Figure S2b,d), providing direct evidence for the co‐localization of Pt, Cu, and Ru at catalytically active surface sites [25, 26]. Collectively, these results validate the successful synthesis of a well‐defined ternary PtCuRu alloy without detectable elemental segregation.

XPS analyses of as‐prepared PtCuRu‐0.05 (Figure 1g; Figure S3) confirm the presence of Pt, Cu and Ru. As shown in the Pt 4*f* XPS spectra (Figure 1g), the prominent peak at 71.3 eV and the weaker peak at 72.1 eV are assigned to Pt^0^ and Pt^2+^ species, respectively [27, 28]. Similarly, in the Cu 2*p* XPS spectra (Figure S3a), the Cu^0^ 2*p*_3/2_ and Cu^2+^ 2*p*_3/2_ peaks are identified at 932.4 and 933.4 eV, respectively [29, 30]. In contrast, the Ru 3*p* XPS spectra exhibit a relatively poor signal‐to‐noise ratio due to the trace doping level; consequently, no deconvolution was performed (Figure S3b). Notably, as‐prepared PtCuRu‐0.05 and as‐prepared PtCu display identical Pt 4*f*_7/2_ and Cu 2*p*_3/2_ binding energies, suggesting negligible electronic modulation upon the introduction of trace Ru. Combined with the XRD findings, it can be concluded that trace Ru doping induces no significant alterations in either the crystal lattice or the electronic configuration.

### 2.2 Core‐Shell Structure With Trace Ru‐Doped Pt‐Rich Surface

An electrochemical dealloying strategy was employed to construct the PtCuRu‐0.05@PtRu core‐shell structure with trace Ru‐doped Pt‐rich surface. The as‐prepared PtCuRu‐0.05 alloy was drop‐cast onto a 3 mm‐diameter glassy carbon electrode and subjected to 30 cyclic voltammetry (CV) cycles in N_2_‐saturated 0.1 M HClO_4_. The cycling was performed at a scan rate of 250 mV/s within a potential window of ‐0.2 to 1.0 V (vs. Ag/AgCl). This process facilitates the selective leaching of unstable surface Cu species and induces structural rearrangement to form a trace Ru‐doped Pt‐rich shell [31]. As shown in Figure S4, the initial CV cycle exhibited distinct redox peaks between 0.5 and 0.6 V vs. RHE, which are assigned to Cu dissolution and partial redeposition [32]. With successive CV cycling, these Cu‐related peaks gradually diminished and eventually stabilized after 30 cycles, signifying the successful removal of unstable surface Cu. Notably, a pair of subtle redox peaks emerged around 0.8 V (vs. RHE) after 10 cycles, attributable to the Ru/Ru(OH)_3_ redox couple [33, 34]. The observation that the anodic peak decreases while the cathodic peak stabilizes during subsequent scans suggests that surface reconstruction constrains surface Ru oxidation, while allowing for the reduction and redeposition of oxidized Ru species. Consequently, this electrochemical dealloying protocol yields a catalyst comprising a PtCuRu alloy core encapsulated by trace Ru‐doped Pt‐rich shell, designated as PtCuRu‐0.05@PtRu.

**FIGURE 2:**
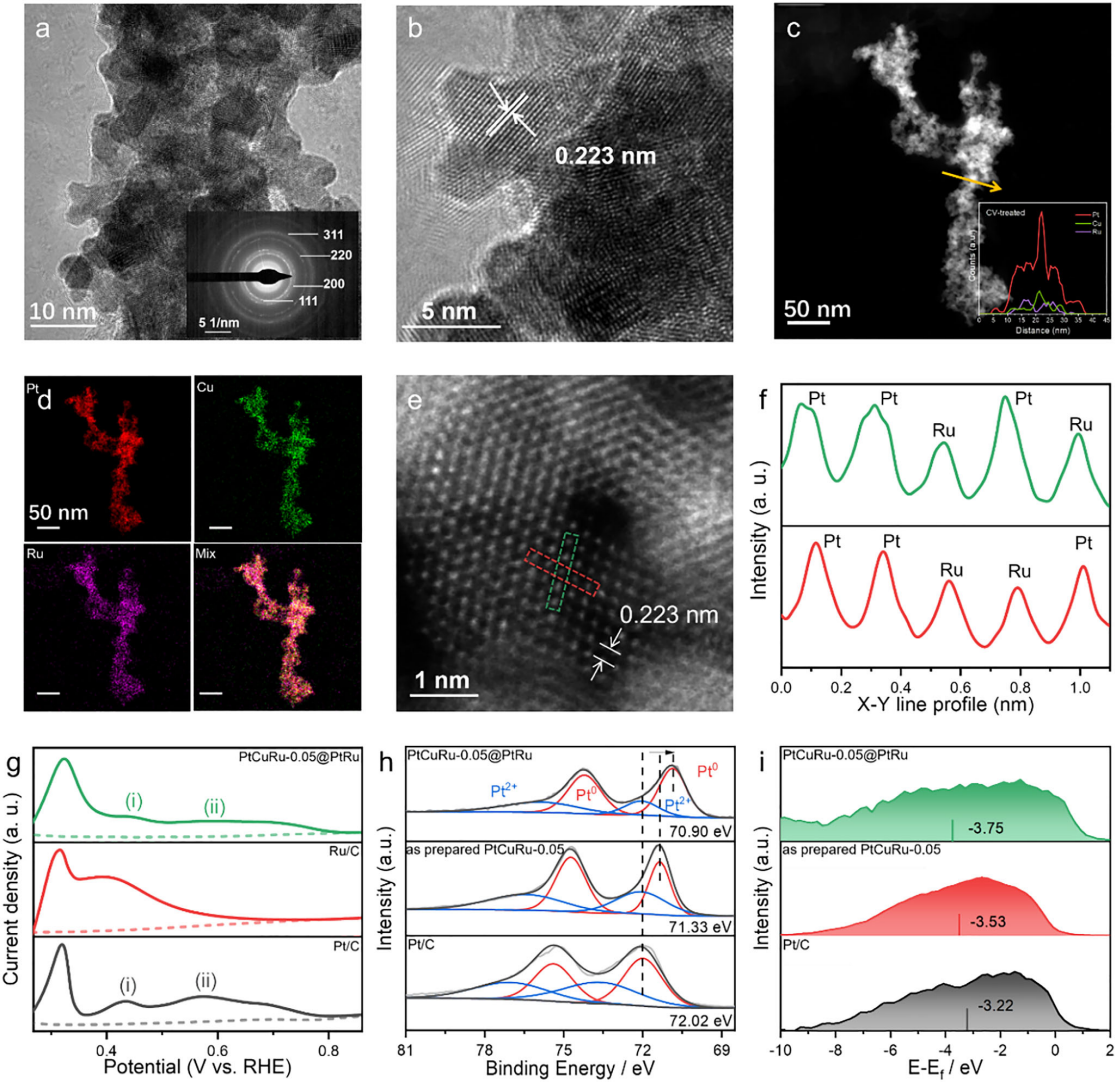
Characterization of PtCuRu‐0.05@PtRu catalyst. (a) HRTEM image (inset shows the corresponding SAED pattern) and (b) lattice spacing analysis of PtCuRu‐0.05@PtRu catalyst. (c) HAADF‐STEM image (inset shows the corresponding EDS line‐scan analysis along the yellow arrow direction), and (d) EDS elemental mapping of PtCuRu‐0.05@PtRu catalyst. (e) Atomic‐resolution HAADF‐STEM image. (f) Intensity line profiles from the atomic‐resolution HAADF‐STEM image (e). (g) Cu_upd_ stripping curves of PtCuRu‐0.05@PtRu, Ru/C and Pt/C. (h) Pt 4*f* XPS spectra and (i) VB‐XPS spectra of PtCuRu‐0.05@PtRu, PtCuRu‐0.05, and commercial Pt/C samples.

The evolution of morphology and composition during the transition from the as‐prepared PtCuRu‐0.05 alloy to the dealloyed PtCuRu‐0.05@PtRu core‐shell structure catalyst was systematically investigated. HRTEM image (Figure 2a) shows that the cross‐linked grains aggregate after electrochemical dealloying, which is attributed to surface atomic rearrangement [35]. The corresponding SAED patterns exhibit sharper and more discrete diffraction rings compared to the as‐prepared alloy, indicating enhanced crystallinity driven by this structural evolution. Crucially, the interplanar spacing of the (111) plane increases from 0.219 nm in the initial as‐prepared alloy to 0.223 nm in the final core‐shell structure catalyst (Figure 2b). While this expansion reflects the selective leaching of smaller Cu atoms from the lattice, the spacing remains notably contracted relative to pristine fcc Pt (0.226 nm). The lattice mismatch between the trace Ru‐doped Pt‐rich shell and the PtCuRu alloy core gives rise to a persistent compressive strain within the shell [36]. The compressive strain is expected to shift the *d*‐band center of the surface Pt atoms, thereby optimizing the adsorption energies of reaction intermediates [37, 38].

HAADF‐STEM and the corresponding EDS mapping (Figure 2c,d) confirm significant compositional changes: the Cu content decreased from 53.6% to 22.5%, while the Pt and Ru contents rose from 43.2% and 3.2% to 67.3% and 10.2%, respectively (Table S2). EDS line‐scan analysis along the yellow arrow in Figure 2c demonstrates the formation of trace Ru‐doped Pt‐rich shell. The Ru signal in the shell is indiscernible due to its trace doping level. The formation of Pt‐rich shell was corroborated through CO stripping voltammetry. Quantitative integration of the CO oxidation peak yielded an ECSA_CO_/ECSA_Hupd_ ratio of 1.57 (Table S3), which definitively demonstrates Pt surface enrichment according to established structure‐property correlations [20, 22, 39, 40, 41, 42, 43]. Atomic‐resolution HAADF‐STEM imaging (Figure 2e; Figure S5a) resolves elemental‐specific Z‐contrast variations within atomic columns (Figure 2f; Figure S5b), directly visualizing Pt‐dominated lattice frameworks with trace Ru dopant at surface sites.

Comparative analysis reveals significant surface reconstruction. Dealloyed PtCuRu‐0.05@PtRu exhibits complete Cu depletion from the outermost layer relative to the as‐prepared precursor. To quantitatively probe surface composition, we employed Cu underpotential deposition (Cu_upd_)—a technique leveraging the distinct underpotential deposition characteristics of Cu on Pt/Ru surfaces under controlled potentials. As Figure 2g demonstrates, the diagnostic peak ratio (i)/(ii) shifts from <1 on pristine Pt to >1 on PtCuRu‐0.05@PtRu. This inversion precisely matches the electrochemical fingerprint of Ru‐modified Pt surfaces, unambiguously verifying the presence of Ru on the catalyst surface [44, 45]. These results collectively demonstrate the successful formation of a PtCuRu‐0.05@PtRu core‐shell structure, inducing compressive strain effect within the Pt‐rich shell.

To further investigate the evolution of the surface electronic structure during the transition to the PtCuRu‐0.05@PtRu core‐shell structure, XPS analysis was performed on the catalysts before and after dealloying. As shown in Figure 2h, the Pt 4*f* spectrum of the PtCuRu‐0.05@PtRu catalyst exhibits primary peaks at 70.90 eV (Pt 4*f*_7/2_). Notably, the Pt 4*f*_7/2_ binding energy of the dealloyed catalyst displays a 0.4 eV negative shift relative to the as‐prepared PtCuRu‐0.05 alloy (71.33 eV), and remains lower than that of commercial Pt/C (72.02 eV). This downward shift indicates increased electron density around the surface Pt atoms, which is attributed to the compressive strain induced by the core‐shell structure [46]. Furthermore, quantitative deconvolution (Figure S6) reveals that the metallic Ptb ^0^ content increases from 54.7% in the as‐prepared PtCuRu‐0.05 alloy to 67.2% after dealloying. This enrichment of metallic Pt species suggests the formation of more active sites, benefiting the MOR activity [47].

The modification of electronic states was further elucidated through valence band‐XPS (VB‐XPS) spectra (Figure 2i). The detailed calculation method can be found in the Experimental Methods section. Compared to commercial Pt/C (‐3.22 eV), both the as‐prepared PtCuRu‐0.05 alloy and the dealloyed catalyst exhibit a downshift in their *d*‐band centers. Specifically, the *d*‐band center of PtCuRu‐0.05@PtRu (‐3.75 eV) shifts more significantly downward than that of the as‐prepared PtCuRu‐0.05 (‐3.53 eV). This additional downshift is primarily attributed to compressive strain within the Pt‐rich shell, arising from the lattice mismatch between the shell and the alloy core that possesses a smaller lattice parameter. According to the *d*‐band center theory, this downshift effectively weakens the adsorption strength of poisoning intermediates (e.g., *CO), thereby optimizing the reaction energetics and enhancing the overall MOR activity [48].

### 2.3 Electrocatalytic Performance Measurements

**FIGURE 3:**
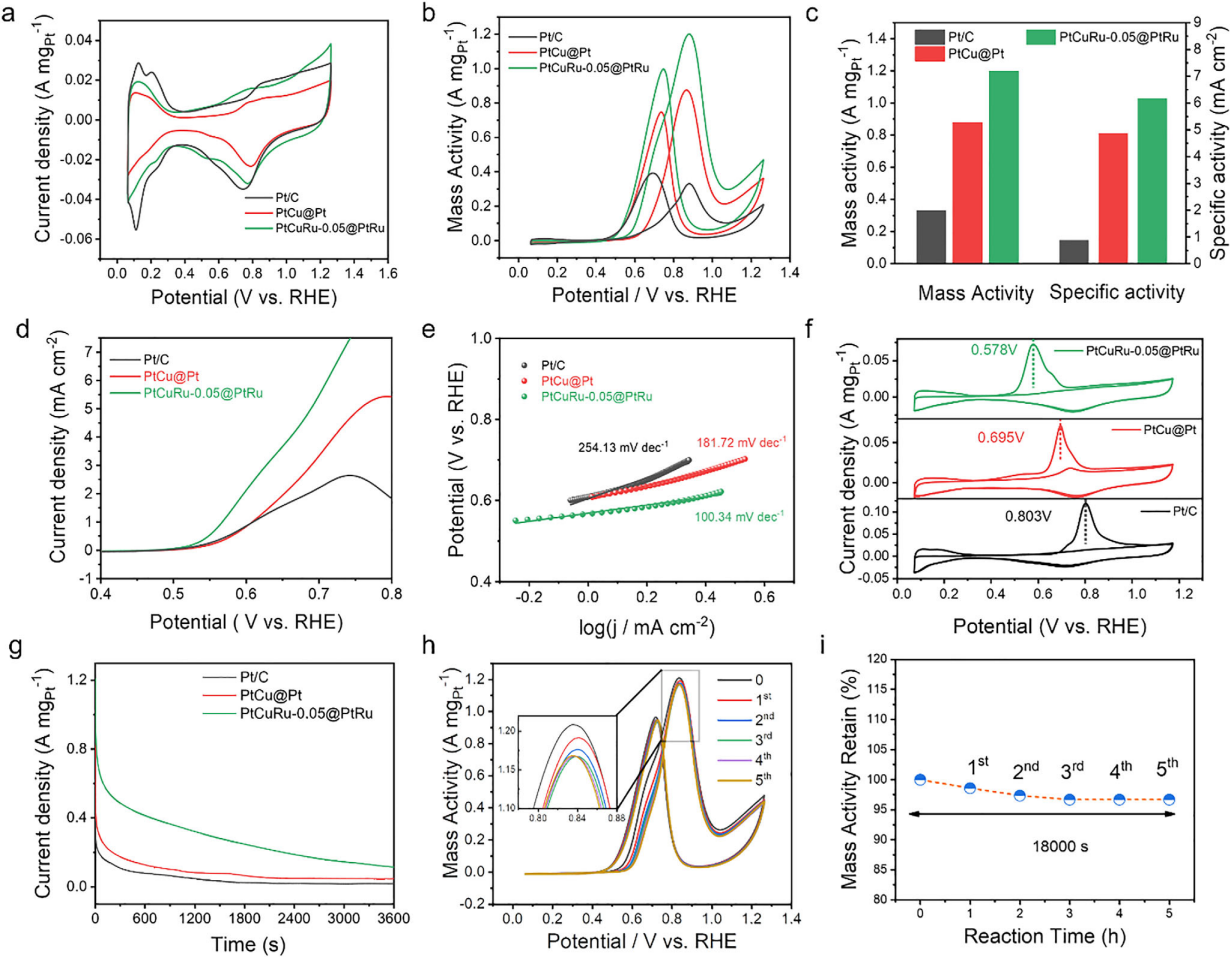
Electrocatalytic performance measurements of PtCu@Pt, PtCuRu‐0.05@PtRu, and commercial Pt/C in the 0.1 M HClO_4_ and 0.5 M CH_3_OH. (a) CV curves. (b) Pt mass‐normalized MOR curves. (c) Bar charts of MA and SA. (d) LSV curves. (e) Tafel plots. (f) CO stripping curves. (g) I‐t curves from the chronoamperometric tests. (h) MOR curves after multi‐cycle chronoamperometric tests. (i) Line plots comparing MOR performance after each chronoamperometric test.

The MOR electrocatalytic performance of the PtCu@Pt and PtCuRu‐x@PtRu (x = 0.025, 0.05, 0.1) core‐shell catalysts was systematically evaluated in acidic media, using commercial Pt/C as a benchmark. To ensure structural consistency, all catalysts underwent identical electrochemical dealloying protocol prior to testing. CV profiles in 0.1 M HClO_4_ (Figure 3a; Figure S7a) exhibited distinct hydrogen adsorption/desorption features, enabling ECSA_Hupd_ calculations. Among the core‐shell catalysts, PtCuRu‐0.05@PtRu achieved the highest ECSA_Hupd_ (19.44 m^2^ g_Pt_^−1^), though still lower than Pt/C (37.75 m^2^ g_Pt_^−1^), primarily attributed to larger particle sizes of dealloyed core‐shell structures. The PtCuRu‐0.05@PtRu catalysts exhibited the highest mass activity (MA) of 1.208 A mg_Pt_^−1^, which is 1.4, 1.2, 1.1, and 3.6 times that of PtCu@Pt (0.875 A mg_Pt_^−1^), PtCuRu‐0.025@PtRu (1.046 A mg_Pt_^−1^), PtCuRu‐0.1@PtRu (1.121 A mg_Pt_^−1^), and commercial Pt/C (0.332 A mg_Pt_^−1^), respectively (Figure 3b,c; Figure S7b). To assess intrinsic catalytic efficiency, specific activity (SA) was derived by normalizing MA against ECSA. Consistently, PtCuRu‐0.05@PtRu demonstrated the highest SA of 6.213 mA cm^−2^, outperforming PtCu@Pt (4.834 mA cm^−2^), PtCuRu‐0.025@PtRu (5.784 mA cm^−2^), PtCuRu‐0.1@PtRu (5.741 mA cm^−2^), and commercial Pt/C (0.879 mA cm^−2^) (Figure S7c,d). Notably, the SA of PtCuRu‐0.05@PtRu represents a 7.1‐fold enhancement over that of commercial Pt/C. This exceptional MA and SA synergy originates from Ru‐dopant‐induced electronic modulation coupled with compressive strain.

To elucidate the origin of enhanced activity, reaction kinetics and CO tolerance were investigated. LSV curves (Figure 3d) revealed that PtCuRu‐0.05@PtRu exhibits a more negative MOR onset potential compared to PtCu@Pt and commercial Pt/C, indicating a reduced energy barrier for methanol oxidation. Consistently, PtCuRu‐0.05@PtRu exhibited the lowest Tafel slope (100.34 mV dec^−1^) compared to PtCu@Pt (181.72 mV dec^−1^) and commercial Pt/C (254.13 mV dec^−1^), confirming accelerated reaction kinetics (Figure 3e) [49, 50]. Electrochemical kinetics analysis revealed PtCuRu‐0.05@PtRu yielded the highest charge‐transfer coefficient (α) quantified from Tafel slopes (Figure S8), indicative of accelerated interfacial electron transfer [7, 50]. Correspondingly, this catalyst achieved the maximum intrinsic turnover frequency (TOF), conclusively establishing its superior catalytic activity among the series (Figure S8) [51].

CO stripping voltammetry further showed a significantly negative‐shifted CO oxidation peak for PtCuRu‐0.05@PtRu (0.578 V) compared to PtCu@Pt (0.695 V) and Pt/C (0.803 V) (Figure 3f). This enhancement arises from dual mechanisms: (1) compressive strain in the Pt‐rich shell downshifts the d‐band center, weakening CO adsorption [52]; (2) trace Ru incorporation promotes *OH generation via facilitated water dissociation at low potentials, enabling efficient oxidative removal of *CO intermediates through a bifunctional mechanism [53]. Thus, the superior MOR activity of PtCuRu‐0.05@PtRu primarily stems from its enhanced reaction kinetics and CO tolerance. CO stripping voltammetry quantified the electrochemically active surface area (ECSA_CO_), revealing that PtCuRu‐0.05@PtRu achieved 30.53 m^2^ g_Pt_^−1^, higher than that of PtCu@Pt (Table S3). Crucially, *in‐situ* attenuated total reflection surface‐enhanced infrared reflection absorption spectroscopy (ATR‐SEIRAS) analysis demonstrated distinct CO tolerance mechanisms. While PtCu@Pt exhibited a diagnostic adsorbed CO (CO_ad_) vibration band at 2095 cm^−1^ (characteristic of linearly‐adsorbed CO intermediates during methanol decomposition, Figure S9a) [54], this fingerprint signature was completely absent on PtCuRu‐0.05@PtRu (Figure S9b). This spectroscopic evidence conclusively established that trace Ru suppressed CO_ad_ poisoning by blocking intermediate formation, thereby promoting the MOR process.

The long‐term durability of the catalysts was assessed using chronoamperometry (CA). As shown in Figure 3g, PtCuRu‐0.05@PtRu maintained the highest steady‐state current density after 3600 s, demonstrating superior stability over PtCu@Pt and commercial Pt/C. Practical viability was further examined through multi‐cycle CA testing with periodic methanol‐containing electrolyte replenishment (Figure S10). Remarkably, after five consecutive 3600 s CA cycles (18,000 s total), the catalyst retained >96% of its initial mass activity (>1.168 A mg_Pt_^−1^) following CV reactivation (Figure 3h,i). This exceptional stability is attributed to the robust anti‐poisoning capability provided by the Ru‐doped shell and the structural integrity of the core‐shell structure. The combination of high intrinsic activity and rapid activity regeneration highlights the potential of PtCuRu‐0.05@PtRu for practical application in DMFCs.

**FIGURE 4:**
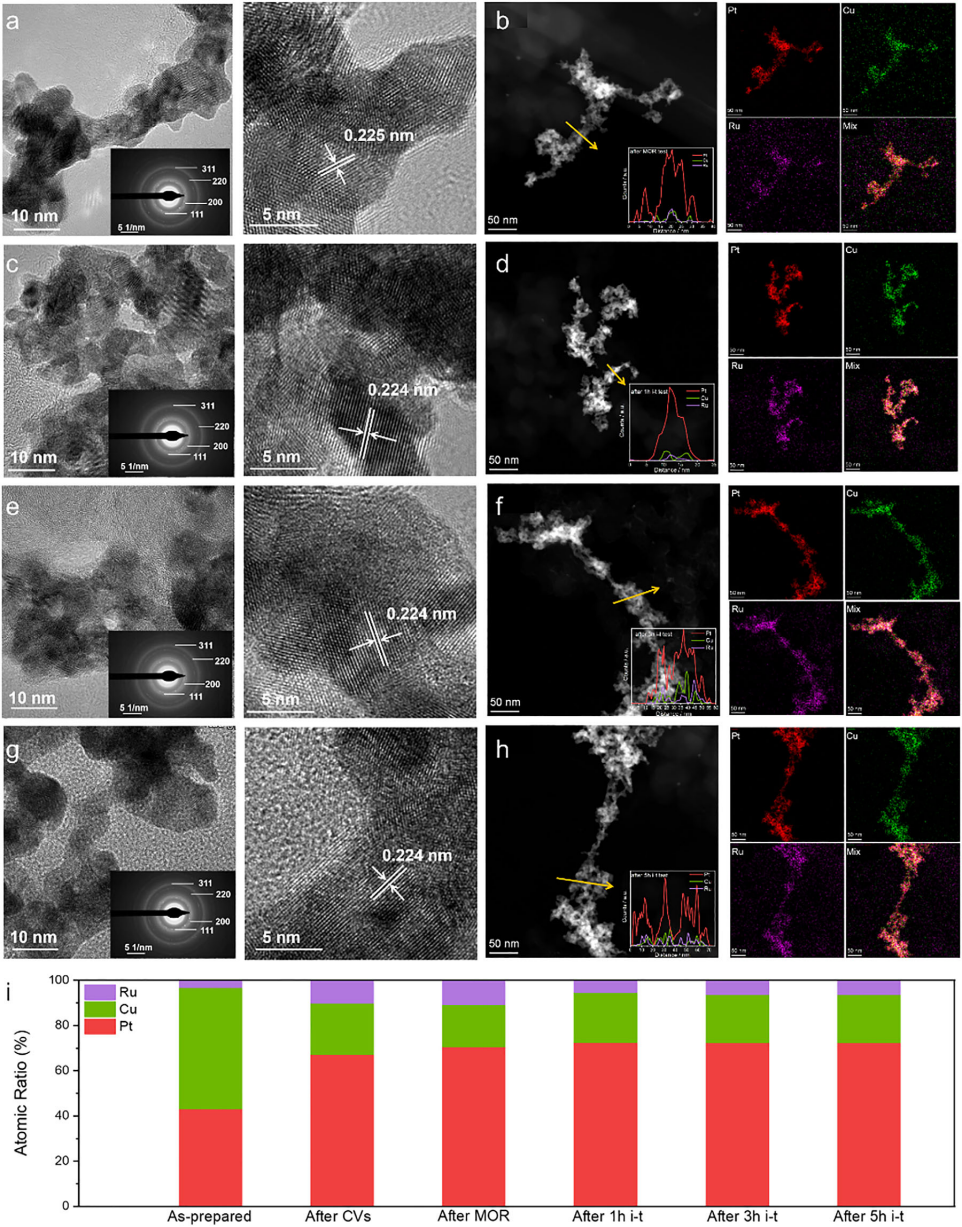
Post‐electrocatalysis characterization of PtCuRu‐0.05@PtRu. (a, b) after MOR. (c, d) after 1 h i‐t. (e, f) after 3 h i‐t. (g, h) after 5 h i‐t. Panels a, c, e, and g show HRTEM images with inset SAED patterns and lattice‐spacing analysis; panels b, d, f, and h show HAADF‐STEM images with EDS line‐scan profiles and EDS elemental mappings. (i) Changes in Pt, Cu, and Ru composition ratios after different electrochemical tests.

The structural evolution of PtCuRu‐0.05@PtRu after extended stability testing (1, 3, and 5 h) was systematically investigated (Figure 4a–h). HRTEM analysis (Figure 4a,c,e,g) reveals highly preserved (111) lattice planes with consistent spacing (0.224 nm) across all post‐test samples. This value is nearly identical to that of the pristine dealloyed catalyst (0.223 nm) and remains significantly contracted relative to pure fcc Pt (0.226 nm). Corresponding SAED patterns maintain sharp discrete diffraction rings, confirming robust retention of crystallinity and (111)‐oriented fcc facets (Figure 4b,d,f,h)—a critical feature for sustained catalytic activity [55, 56]. This lattice stability confirms that the compressive strain within the Pt‐rich shell is effectively preserved, ensuring that the strain‐induced electronic modulation of the active Pt sites remains functional during prolonged operation.

## 3 Conclusion

In conclusion, we successfully synthesized trace Ru‐doped PtCuRu‐0.05@PtRu core‐shell electrocatalyst that demonstrates exceptional activity and durability for the MOR in acidic media. The catalyst's superior performance originates from synergistic compressive strain and bifunctional effects: lattice mismatch between the ternary PtCuRu core and trace Ru‐doped Pt‐rich shell induces persistent compressive strain, downshifting the Pt d‐band center to weaken *CO intermediate binding. Simultaneously, trace Ru dopants facilitate low‐potential water dissociation, generating *OH species that oxidatively remove *CO. Beyond intrinsic activity, the Pt‐rich shell acts as a physical barrier against electrolyte corrosion, ensuring structural and compositional integrity. Electrochemical validation confirms a record mass activity of 1.208 A mg_Pt_^−1^ and retains approximately over 96% of its initial performance after 18,000 s of cumulative operation, demonstrating compressive strain coupled with trace surface doping as a robust strategy for high‐performance fuel cell applications.

## 4 Experimental Methods

### 4.1 Chemicals

The reagents required for the experiments were analytical grade. Chloroplatinic acid (H_2_PtCl_6_, 99.95%), ruthenium chloride (RuCl_3_·xH_2_O, 99.95%) and copper (II) sulfate pentahydrate (CuSO_4_·5H_2_O, 99.8%) were purchased from Adamas‐beta. Cupric chloride dihydrate (CuCl_2_·2H_2_O, 98%), potassium bromide (KBr, ≥ 99%) and cetyltrimethylammonium chloride (CTAC, ≥ 97%) were purchased from Aladdin. Ascorbic acid (AA_,_ 98%), sulfuric acid (H_2_SO_4_, 95.0∼98.0%) and perchloric acid (HClO_4_, 70.0∼72.0%) were obtained from Sinopharm. Absolute methanol (CH_3_OH, ≥99.5%) was obtained from General‐Reagent. Carbon black (Vulcan XC‐72) was purchased from Cabot. Nafion (5 wt.%) was purchased from Dupont. A commercial Pt/C (20 wt.%) catalyst was obtained from Johnson Matthey (London, UK). A commercial Ru/C (20 wt.%) catalyst was obtained from Macklin. Deionized water (DIW, 18.2 MΩ cm^−1^) was employed for the preparation of all solutions. All reagents were used without any further purification.

### 4.2 Synthesis of Core‐Shell PtCuRu@PtRu Electrocatalysts

The as‐prepared PtCuRu‐x (x = 0, 0.025, 0.05, 0.1) catalysts were synthesized via a one‐pot liquid‐phase reduction method. H_2_PtCl_6_, CuCl_2_·2H_2_O, and RuCl_3_·xH_2_O were employed as precursors for Pt, Cu, and Ru, respectively, with AA as the reducing agent, and KBr and CTAC as structural directing agents. In a typical synthesis, 26 mg of CTAC was dissolved in 5 mL of deionized water in a 50 mL flask under ultrasonication. Subsequently, 0.5 mL of 10 mM H_2_PtCl_6_, 0.5 mL of 10 mM CuCl_2_·2H_2_O, 0.025 mL of 20 mM RuCl_3_·xH_2_O, and 0.5 mL of 10 mM KBr were sequentially added to the CTAC solution under magnetic stirring at 50°C. After 5 min, 0.5 mL of AA solution was introduced, and the reaction proceeded for 1 h. The resulting product was centrifuged and washed three times with ethanol/water mixtures to remove impurities, followed by dispersion in ethanol for subsequent characterization or electrochemical testing. By adjusting the volume of RuCl_3_·xH_2_O (0, 0.012, or 0.05 mL), PtCu, PtCuRu‐0.025, and PtCuRu‐0.1 were prepared.

To construct the core‐shell PtCuRu‐x@PtRu (x = 0, 0.025, 0.05, 0.1) catalysts, an electrochemical dealloying strategy was employed. The process was conducted in a three‐electrode system (CH Instruments, Shanghai) using a Pt counter electrode, Ag/AgCl reference electrode, and a polished glassy carbon electrode (GCE, 3 mm diameter) as the working electrode. Catalyst inks of as‐prepared PtCuRu‐x (x = 0.025, 0.05, 0.1) and PtCu were drop‐cast onto the GCE surface and dried at room temperature, with Pt loadings controlled between 10‐15 µg/cm^2^. Electrochemical dealloying was performed in N_2_‐saturated 0.1 M HClO_4_ by cyclic voltammetry (CV) between ‐0.2 and 1.0 V (vs. Ag/AgCl) at a scan rate of 250 mV/s. This process selectively etched unstable surface Cu atoms, yielding PtCuRu‐x@PtRu catalysts with trace Ru‐doped Pt‐rich shell. The Pt‐rich shell not only enhances stability by shielding the core from electrolyte corrosion but also optimizes electronic effects for improved catalytic performance.

### 4.3 Characterizations

Transmission electron microscopy (TEM) and high‐resolution transmission electron microscopy (HRTEM) were performed using FEI Talos F200X. Scanning TEM (STEM), STEM‐energy dispersive X‐ray spectroscopy (STEM‐EDS) line‐scanning, and elemental mapping characterization were used to further confirm the nanostructures, morphologies, and elemental distributions of the catalysts (FEI Talos F200X). Atomic‐resolution high‐angle annular dark‐field‐scanning transmission electron microscopy (Atomic‐resolution HAADF‐STEM) data were obtained by the Thermal Fisher Themis Z with spherical aberration correctors. X‐ray diffraction (XRD) patterns of the synthesized catalysts were acquired by using a Rigaku SmartLab SE with Cu Kα as the X‐ray source (40 mA, 40 kV, λ= 0.15406 nm). X‐ray photoelectron spectroscopy (XPS) tests (Thermo Scientific K‐Alpha with monochromatic Al Kα X‐ray source) were used to collect the XPS spectra of the catalysts. Synchrotron X‐ray absorption spectroscopy spectra were collected at the BL14W1 beamline of the Shanghai Synchrotron Radiation Facility (SSRF), and the corresponding data were analyzed using Athena software (ver. 0.9.26). The extended X‐ray absorption fine structure (EXAFS) data fitting was performed using Artemis software. Agilent 5800 ICP‐OES Inductively Coupled Plasma Optical Emission Spectrometry (ICP‐OES), Agilent (US), 5800 ICP‐OES was used. *In‐situ* attenuated total reflection surface‐enhanced infrared reflection absorption spectroscopy (ATR‐SEIRAS) measurements were conducted with a Thermal Fisher Nicolet iS20 equipped with a liquid nitrogen‐cooled MCT detector.

### 4.4 Electrochemical Measurements

Before the electrocatalytic measurements, the homogeneous catalyst ink of electrocatalysts was fabricated by ultrasonically mixing 990 µL ethanol dispersion of the catalysts,10 µL 5 wt.% Nafion solution, and 1 mg carbon powder for about 30 min. For the electrochemical tests, the 5 µL of ink was dropped onto a clean glassy‐carbon electrode (GCE, diameter: 3 mm) to prepare the working electrode, and an Ag/AgCl (3.5 M KCl) and a Pt foil (1cm×1 cm) served as reference and counter electrodes, respectively. The Pt loading of all catalyst samples on the GCE surface was precisely controlled at 11 µg cm^−2^. All the electrochemical measurements were performed on a CHI 760E (Chenhua, Shanghai) electrochemical workstation with a typical three‐electrode configuration at ambient temperature, and all the recorded potentials were converted to the reversible hydrogen electrode (RHE). The cyclic voltammograms (CVs) were collected in N_2_‐saturated 0.1 M HClO_4_ from ‐0.2 to 1.0 V (vs. Ag/AgCl) with a scan rate of 50 mV s^−1^. The electrochemical active surface area (ECSA) was estimated according to the underpotentially deposited H (H_upd_) methods. From the charge of the H_upd_ desorption peak in the recorded CVs, the ECSA of catalysts were determined from one monolayer of hydrogen desorption on Pt with a criterion of 0.21 mC cm^−2^. The ECSA obtained from CO stripping was calculated by integrating the CO oxidation charge, where the oxidation charge of one monolayer of CO is 0.42 mC cm^−2^.

Cu underpotential deposition (Cu_upd_) was used to analyze the atomic composition of catalyst samples surface. An Ag/AgCl and a graphite rod were used as the reference and counter electrodes, respectively. A GCE coated with the catalyst samples was used as the working electrode. The background current was initially recorded in N_2_‐saturated 50 mM H_2_SO_4_ from ‐0.2 to 1.0 V (vs. Ag/AgCl) with a scan rate of 10 mV s^−1^. Then, the electrode was immediately transferred into N_2_‐saturated 2 mM CuSO_4_ + 50 mM H_2_SO_4_ solution and kept at 0.13 V (vs. Ag/AgCl) for 3 min. In Cu‐stripping measurement, the electrode was immersed in N_2_‐saturated 2 mM CuSO_4_ + 50 mM H_2_SO_4_ solution, and the potential was positively swept from ‐0.2 to 1.0 V (vs. Ag/AgCl) with a scan rate of 10 mV s^−1^.

The methanol oxidation reaction (MOR) polarization curves were obtained at the scan rate of 50 mV s^−1^ in N_2_‐saturated 0.1 M HClO_4_ and 0.5 M methanol solution. Before MOR tests, the catalysts were activated by conducting the CVs to be stable in N_2_‐saturated 0.1 M HClO_4_ at a sweep rate of 250 mV s^−1^. The chronoamperometry (CA) measurements of MOR were conducted in 0.1 M HClO_4_ and 0.5 M methanol solution at 0.6 V vs. Ag/AgCl. For the CO stripping measurement, the working electrode was held at a constant potential of 0.1 V vs. RHE under a flow of CO bubbled into the N_2_‐saturated 0.1 M HClO_4_ electrolyte for 10 min and then changed CO to N_2_ for another 20 min to absorb monolayer CO molecules. Afterward, the working electrode was recorded for two cycles at a scan rate of 50 mV s^−1^.

### 4.5 Electrochemical In‐Situ ATR‐SEIRAS Experiments

In situ ATR‐SEIRAS measurements were performed on a Fourier transform infrared spectrometer equipped with an MCT/A detector with a resolution of 4 cm^−1^. The test was conducted in a spectroelectrochemical cell with a three‐electrode configuration. The Au film was plated on a Si prism, which played a role in enhancing signals. The catalyst ink was sprayed onto the Au film. Ag/AgCl and Pt wire were used as the reference and counter electrodes, respectively. The spectra were collected from 0 to 1.0 V vs. RHE in N_2_‐saturated 0.1 M HClO_4_ + 0.5 M methanol.

### 4.6 The Calculation Method of d‐Band Centers

The d‐band centers of PtCuRu‐0.05@PtRu, as prepared PtCuRu‐0.05 and commercial Pt/C for the valence band spectra were determined as

(1) $$\begin{array}{c} \int N\left(\varepsilon \right)\varepsilon d\varepsilon /\int N\left(\varepsilon \right)d\varepsilon \;\# \end{array}$$

In the range 2 ∼‐10 eV, where *N*(ε) is the density of states [57, 58, 59].

### 4.7 The Calculation Method of Charge Transfer Coefficient

Tafel slope of all studied MOR electrocatalysts was determined from the Tafel plots, which were fitted by the Tafel equation on the following:

(2) $$\begin{array}{c} \eta =blogj+a=\frac{2.3RT}{\alpha nF}logj-\frac{2.3RT}{\alpha nF}logj_{0}\;\# \end{array}$$

where *R* and *T* (K) present gas constant and absolute temperature, respectively; α denotes a charge transfer coefficient, *F* is a Faraday constant, and *j*_0_ is an exchange current density.

### 4.8 The Calculation Method of TOF

The TOF calculation method is as followed:

(3) $$\begin{array}{c} TOF=\frac{I}{znF}\;\# \end{array}$$

where *TOF* is the turnover frequency (s^−1^), *I* is the current (A), *z* is the number of electrons transferred in the reaction (For MOR, *z* = 1), n is the number of moles of the active sites (mol), *F* is the Faraday constant. The number of moles of the active sites can be determined by H_upd_ method:

(4) $$\begin{array}{c} n=\frac{N}{N_{A}}=\frac{Q_{Hupd}/e}{N_{A}}\;\# \end{array}$$

where *Q_Hupd_* is the charges obtained from H_upd_, *e* is the elementary charge, *N_A_* represents the Avogadro constant.

## Conflicts of Interest

The authors declare no conflicts of interest.

## Supporting information




**Supporting File**: advs75638‐sup‐0001‐SuppMat.docx.

## Data Availability

The data that supports the findings of this study are available in the supplementary material of this article.

## References

[1] J. Wang , B. Zhang , W. Guo , et al., “Toward Electrocatalytic Methanol Oxidation Reaction: Longstanding Debates and Emerging Catalysts,” Advanced Materials 35, no. 26 (2023): 2211099, 10.1002/adma.202211099. 36706444

[2] L. Yang , J. J. Ge , C. P. Liu , G. L. Wang , and W. Xing , “Approaches to Improve the Performance of Anode Methanol Oxidation Reaction—A Short Review,” Current Opinion in Electrochemistry 4, no. 1 (2017): 83–88, 10.1016/j.coelec.2017.10.018.

[3] A. M. Jasim , G. Xu , M. J. Young , and Y. Xing , “Tuning Vacancy in Metal Oxide Support to Enhance Activity and Durability of Pt Catalysts for the Methanol Oxidation Reaction,” ACS Catalysis 15, no. 5 (2025): 4350–4358, 10.1021/acscatal.5c00259.

[4] A. R. Poerwoprajitno , L. Gloag , J. Watt , et al., “A Single‐Pt‐Atom‐on‐Ru‐Nanoparticle Electrocatalyst for CO‐Resilient Methanol Oxidation,” Nature Catalysis 5, no. 3 (2022): 231–237, 10.1038/s41929-022-00756-9.

[5] Y. Nie , Z. Li , Y. Wang , et al., “The 5d‐5p‐3d Orbital Hybridization Induced by Light Incorporation of Cu into Surface‐uneven Pt3Sn Intermetallic Nanocubes Customizes Dual‐intermediates Adsorptions for CO‐resilient Methanol Oxidation,” Applied Catalysis B: Environmental 343 (2024): 123494, 10.1016/j.apcatb.2023.123494.

[6] Z. Li , S. Ke , X. Zheng , et al., “Modulating D‐orbital Electronic Configuration of PtRu Via Charge Donation from Co‐Enriched Core Boosts Methanol Electrooxidation,” Chemical Engineering Journal 493, no. 1385–8947 (2024): 152544, 10.1016/j.cej.2024.152544.

[7] S. Xing , Z. Liu , Y. Jiang , et al., “Platinum–copper Nanowire Networks with Enhanced CO Tolerance Toward Methanol Oxidation Electrocatalysis,” Chemical Science 16 (2025): 9311–9319, 10.1039/d5sc00656b. 40290339 PMC12022767

[8] Y. Deng , H. Liu , L. Lai , et al., “Platinum‐Ruthenium Bimetallic Nanoparticle Catalysts Synthesized via Direct Joule Heating for Methanol Fuel Cells,” Small 21 (2024): 2403967, 10.1002/smll.202403967. 39106223 PMC11840475

[9] Z. Xia , R. Yu , Y. Wang , et al., “Cavities‐Induced Compressive Strain in Unique Nanotubes Boosts the C_1_ Pathway of Ethanol Oxidation Electrocatalysis,” ACS Nano 19, no. 7 (2025): 7379–7390, 10.1021/acsnano.4c18350. 39955788

[10] Y. Sun , S. Zhang , S. Sun , et al., “Cu Tailoring Pt Enables Branched‐Structured Electrocatalysts with High Concave Surface Curvature toward Efficient Methanol Oxidation,” Small 20, no. 18 (2023): 2307970, 10.1002/smll.202307970. 38054785

[11] Q. Yang , S. Zhang , F. Wu , et al., “Efficient and Stable PtFe Alloy Catalyst for Electrocatalytic Methanol Oxidation with High Resistance to CO,” Journal of Energy Chemistry 90 (2024): 327–336, 10.1016/j.jechem.2023.11.038.

[12] T. Xia , K. Zhao , Y. Zhu , et al., “Mixed‐Dimensional Pt–Ni Alloy Polyhedral Nanochains as Bifunctional Electrocatalysts for Direct Methanol Fuel Cells,” Advanced Materials 35, no. 2 (2023): 2206508, 10.1002/adma.202206508. 36281798

[13] M. Zhu , Y. Wang , Y. Wu , et al., “Greatly Enhanced Methanol Oxidation Reaction of CoPt Truncated Octahedral Nanoparticles by External Magnetic Fields,” Energy & Environmental Materials 6, no. 5 (2022): 12403, 10.1002/eem2.12403.

[14] K. Wang , D. Huang , Y. Guan , F. Liu , J. He , and Y. Ding , “Fine‐Tuning the Electronic Structure of Dealloyed PtCu Nanowires for Efficient Methanol Oxidation Reaction,” ACS Catalysis 11, no. 23 (2021): 14428–14438, 10.1021/acscatal.1c04424.

[15] M. Qiao , F. Y. Meng , H. Wu , Y. Wei , X. F. Zeng , and J. X. Wang , “PtCuRu Nanoflowers with Ru‐Rich Edge for Efficient Fuel‐Cell Electrocatalysis,” Small 18, no. 48 (2022): 2204720, 10.1002/smll.202204720. 36269882

[16] Y. Lu , L. Liang , S. Ye , Z. Chen , W. Zhao , and Z. Cui , “Pt_3_Sn_0.5_Mn_0.5_ Intermetallic Electrocatalyst with Superior Stability for CO‐Resilient Methanol Oxidation,” ACS Applied Materials & Interfaces 16, no. 27 (2024): 35134–35142, 10.1021/acsami.4c05906. 38940277

[17] R. Jin , Q. Lu , J. Liao , et al., “Prompt Template‐Free Synthesis of Porous PtPb Sponge‐Like Nanostructure for Electro‐Oxidation of Methanol and Carbon Monoxide,” Electrochimica Acta 508 (2024): 145210, 10.1016/j.electacta.2024.145210.

[18] D. Liu , Y. Zhang , H. Liu , et al., “Acetic Acid‐Assisted Mild Dealloying of Fine CuPd Nanoalloys Achieving Compressive Strain Toward High‐Efficiency Oxygen Reduction and Ethanol Oxidation Electrocatalysis,” Carbon Energy 5, no. 7 (2023): 324, 10.1002/cey2.324.

[19] J. Yoo , Y. Park , J. Choi , et al., “Electrochemical Dealloying of Ni‐Rich Pt–Ni Nanoparticle Network for Robust Oxygen‐Reduction Electrocatalysts,” Acs Sustainable Chemistry & Engineering 11, no. 42 (2023): 15460–15469, 10.1021/acssuschemeng.3c04866.

[20] H. Jin , Z. Xu , Z.‐Y. Hu , et al., “Mesoporous Pt@Pt‐skin Pt_3_Ni Core‐shell Framework Nanowire Electrocatalyst for Efficient Oxygen Reduction,” Nature Communications 14, no. 1 (2023): 1518, 10.1038/s41467-023-37268-4. PMC10024750 36934107

[21] Y. Zhuang , Y. Iguchi , T. Li , et al., “Platinum–Nickel Alloy Nanowire Electrocatalysts Transform into Pt‐Skin Beads‐on‐Nanowires Keeping Oxygen Reduction Reaction Activity during Potential Cycling,” ACS Catalysis 14, no. 3 (2024): 1750–1758, 10.1021/acscatal.3c04709.

[22] S. Yin , Y. Song , H. Liu , et al., “Well‐Defined PtCo@Pt Core‐Shell Nanodendrite Electrocatalyst for Highly Durable Oxygen Reduction Reaction,” Small 21, no. 8 (2025): 2410080, 10.1002/smll.202410080. 39780638

[23] H.‐H. Li , Q.‐Q. Fu , L. Xu , et al., “Highly Crystalline PtCu Nanotubes with Three Dimensional Molecular Accessible and Restructured Surface for Efficient Catalysis,” Energy & Environmental Science 10, no. 8 (2017): 1751–1756, 10.1039/c7ee00573c.

[24] T. He , W. Wang , F. Shi , et al., “Mastering the Surface Strain of Platinum Catalysts for Efficient Electrocatalysis,” Nature 598, no. 7879 (2021): 76–81, 10.1038/s41586-021-03870-z. 34616058

[25] J. Su , J. Wang , Z. Wang , T. K. Kim , and J. Wang , “Local Atom Disorder Mediated Crystal Facet Valence State of PtCu Alloy for Ultrafast Ammonia Oxidation Dehydrogenation,” Advanced Functional Materials 36, no. 20 (2025): 19518, 10.1002/adfm.202519518.

[26] F. Yang , Y. Wang , Y. Cui , et al., “Sub‐3 Nm Pt@Ru Toward Outstanding Hydrogen Oxidation Reaction Performance in Alkaline Media,” Journal of the American Chemical Society 145, no. 50 (2023): 27500–27511, 10.1021/jacs.3c08908. 38056604

[27] P. Kuang , Y. Wang , B. Zhu , et al., “Pt Single Atoms Supported on N‐Doped Mesoporous Hollow Carbon Spheres with Enhanced Electrocatalytic H_2_‐Evolution Activity,” Advanced Materials 33, no. 18 (2021): 2008599, 10.1002/adma.202008599. 33792090

[28] L. Zhao , Z. Zhu , J. Wang , et al., “Robust p‐d Orbital Coupling in PtCoIn@Pt Core‐Shell Catalysts for Durable Proton Exchange Membrane Fuel Cells,” Angewandte Chemie International Edition 64, no. 19 (2025): 202501805, 10.1002/anie.202501805. 40044627

[29] S. Yan , Z. Chen , Y. Chen , et al., “High‐Power CO_2_‐to‐C_2_ Electroreduction on Ga‐Spaced, Square‐Like Cu Sites,” Journal of the American Chemical Society 145, no. 48 (2023): 26374–26382, 10.1021/jacs.3c10202. 37992232

[30] J. Yang , X. Jin , Z. Cheng , et al., “Facile and Green Synthesis of Bifunctional Carbon Dots for Detection of Cu^2+^ and ClO—in Aqueous Solution,” Acs Sustainable Chemistry & Engineering 9, no. 39 (2021): 13206–13214, 10.1021/acssuschemeng.1c03868.

[31] S. Koh and P. Strasser , “Modifying Catalytic Reactivity for Oxygen Reduction by Voltammetric Surface Dealloying,” Journal of the American Chemical Society 129, no. 42 (2007): 12624–12625, 10.1021/ja0742784. 17910452

[32] P. Strasser , S. Koh , and J. Greeley , “n Experimental and DFT Computational Analysis,” Physical Chemistry Chemical Physics 10, no. 25 (2008): 3670–3683, 10.1039/B803717E. 18563228

[33] J. Juodkazytė , R. Vilkauskaitė , B. Šebeka , and K. Juodkazis , “Difference between Surface Electrochemistry of Ruthenium and RuO_2_ Electrodes,” Transactions of the IMF 85, no. 4 (2007): 194–201, 10.1179/174591907X16413.

[34] M. Minichová , T. Priamushko , M. Zlatar , K. J. J. Mayrhofer , and S. Cherevko , “pH Dependence of Noble Metals Dissolution: Ruthenium,” ChemElectroChem 12, no. 9 (2025): 202400651, 10.1002/celc.202400651.

[35] Q.‐Q. Fu , H.‐H. Li , L. Xu , Y.‐D. Li , and S.‐H. Yu , “Electrochemically Activated Surface Reconstruction of PdCu Nanotubes for Improved Ethanol Oxidation Electrocatalysis,” Small Structures 3, no. 6 (2022): 2100216, 10.1002/sstr.202100216.

[36] Z. Zhong , Y. Tu , L. Zhang , et al., “Surface Strain Effect on Electrocatalytic Hydrogen Evolution Reaction of Pt‐Based Intermetallics,” ACS Catalysis 14, no. 5 (2024): 2917–2923, 10.1021/acscatal.3c06291.

[37] M. Liu , S. Zhou , M. Figueras‐Valls , et al., “Compressively Strained and Interconnected Platinum Cones with Greatly Enhanced Activity and Durability toward Oxygen Reduction,” Advanced Functional Materials 34, no. 45 (2024): 2404677, 10.1002/adfm.202404677.

[38] L. Xu , X. Tai , L. Feng , et al., “Pt‐Shell‐Protected PtZn Intermetallic Nanoclusters for Highly Efficient Propane Dehydrogenation,” ACS Catalysis 15, no. 5 (2025): 4172–4184, 10.1021/acscatal.4c07624.

[39] C. Chen , Y. Kang , Z. Huo , et al., “Highly Crystalline Multimetallic Nanoframes with Three‐Dimensional Electrocatalytic Surfaces,” Science 343, no. 6177 (2014): 1339–1343, 10.1126/science.1249061. 24578531

[40] G. M. Leteba , S. L. George , D. R. G. Mitchell , et al., “Synthesis of PtNi Nanoparticles to Accelerate the Oxygen Reduction Reaction,” ChemPlusChem 89, no. 7 (2024): 202400083, 10.1002/cplu.202400083. 38523404

[41] V. Stamenković , T. J. Schmidt , P. N. Ross , and N. M. Marković , “Kinetics of Oxygen Reduction on Well‐Defined Pt_3_Ni and Pt_3_Co Alloy Surfaces,” The Journal of Physical Chemistry B 106, no. 46 (2002): 11970, 10.1021/jp021182h.

[42] D. F. van der Vliet , C. Wang , D. Li , et al., “Unique Electrochemical Adsorption Properties of Pt‐Skin Surfaces,” Angewandte Chemie International Edition 51, no. 13 (2012): 3139–3142, 10.1002/anie.201107668. 22351117

[43] H. Zhu , S. Zhang , D. Su , G. Jiang , and S. Sun , “Surface Profile Control of FeNiPt/Pt Core/Shell Nanowires for Oxygen Reduction Reaction,” Small 11, no. 29 (2015): 3545–3549, 10.1002/smll.201500330. 25786658

[44] C. L. Green and A. Kucernak , “Determination of the Platinum and Ruthenium Surface Areas in Platinum−Ruthenium Alloy Electrocatalysts by Underpotential Deposition of Copper. I. Unsupported Catalysts,” The Journal of Physical Chemistry B 106, no. 5 (2002): 1036–1047, 10.1021/jp0131931.

[45] S. Zhu , X. Qin , F. Xiao , et al., “The Role of Ruthenium in Improving the Kinetics of Hydrogen Oxidation and Evolution Reactions of Platinum,” Nature Catalysis 4, no. 8 (2021): 711–718, 10.1038/s41929-021-00663-5.

[46] H. H. Li , C. H. Cui , S. Zhao , et al., “Mixed‐PtPd‐Shell PtPdCu Nanoparticle Nanotubes Templated from Copper Nanowires as Efficient and Highly Durable Electrocatalysts,” Advanced Energy Materials 2, no. 10 (2012): 1182–1187, 10.1002/aenm.201200207.

[47] Y. Zhang , B. Huang , G. Luo , et al., “Atomically Deviated Pd‐Te Nanoplates Boost Methanol‐tolerant Fuel Cells,” Science Advances 6, no. 31 (2020): aba9731, 10.1126/sciadv.aba9731. PMC7439301 32832686

[48] Q. Feng , S. Zhao , D. He , et al., “train Engineering to Enhance the Electrooxidation Performance of Atomic‐Layer Pt on Intermetallic Pt_3_Ga,” Journal of the American Chemical Society 140, no. 8 (2018): 2773–2776, 10.1021/jacs.7b13612. 29432012

[49] J. Hu , C. Fang , X. Jiang , D. Zhang , and Z. Cui , “Ultrathin and Porous 2D PtPdCu Nanoalloys as High‐Performance Multifunctional Electrocatalysts for Various Alcohol Oxidation Reactions,” Inorganic Chemistry 61, no. 24 (2022): 9352–9363, 10.1021/acs.inorgchem.2c01257. 35674700

[50] T. T. Huynh , Q. Huynh , Q. V. Nguyen , and H. Q. Pham , “Lattice Strain and Composition Effects on the Methanol Oxidation Performance of Platinum–Ruthenium–Nickel Ternary Nanocatalysts,” Inorganic Chemistry 62, no. 49 (2023): 20477–20487, 10.1021/acs.inorgchem.3c03518. 37990435

[51] Y. Zhou , W. Xu , Z. Wei , et al., “Molecular Iridium Catalyzed Electrochemical Formic Acid Oxidation: Mechanistic Insights,” Angewandte Chemie International Edition 64, no. 1 (2025): 202412901, 10.1002/anie.202412901. 39141415

[52] F. Maillard , G. Q. Lu , A. Wieckowski , and U. Stimming , “Ru‐Decorated Pt Surfaces as Model Fuel Cell Electrocatalysts for CO Electrooxidation,” The Journal of Physical Chemistry B 109, no. 34 (2005): 16230–16243, 10.1021/jp052277x. 16853064

[53] C. Liang , J. Yao , N. Gao , et al., “RuO ‐PtZn Catalyst Boosting Methanol Electro‐Oxidation by Synergic Water‐Activation for High‐Performance Direct Methanol Fuel Cell,” Chinese Journal of Catalysis 80 (2026): 304–315, 10.1016/S1872-2067(25)64836-4.

[54] X. Huang , B. Xu , Y. Sun , et al., “Phase‐Rearrangement‐Induced Atomic Replacement toward Customizing Noble‐Metal Intermetallics,” Journal of the American Chemical Society 147, no. 50 (2025): 46449–46460, 10.1021/jacs.5c16486. 41335108

[55] K. Jiang , D. Zhao , S. Guo , et al., “Efficient Oxygen Reduction Catalysis by Subnanometer Pt Alloy Nanowires,” Science Advances 3, no. 2 (2017): 1601705, 10.1126/sciadv.1601705. PMC5325541 28275723

[56] Z. Kong , Y. Maswadeh , J. A. Vargas , et al., “Origin of High Activity and Durability of Twisty Nanowire Alloy Catalysts under Oxygen Reduction and Fuel Cell Operating Conditions,” Journal of the American Chemical Society 142, no. 3 (2020): 1287–1299, 10.1021/jacs.9b10239. 31885267

[57] S. J. Hwang , S.‐K. Kim , J.‐G. Lee , et al., “Role of Electronic Perturbation in Stability and Activity of Pt‐Based Alloy Nanocatalysts for Oxygen Reduction,” Journal of the American Chemical Society 134, no. 48 (2012): 19508–19511, 10.1021/ja307951y. 23131009

[58] D. Liu , Q. Zeng , C. Hu , et al., “Light Doping of Tungsten into Copper‐platinum Nanoalloys for Boosting Their Electrocatalytic Performance in Methanol Oxidation,” Nano Research Energy 1 (2022): 9120017, 10.26599/NRE.2022.9120017.

[59] X. Wu , F. Chen , N. Zhang , et al., “Activity Trends of Binary Silver Alloy Nanocatalysts for Oxygen Reduction Reaction in Alkaline Media,” Small 13, no. 15 (2017): 1603387, 10.1002/smll.201603387. 28151572

