# Supplementary material for: Trace Ru‐Doped PtCuRu@PtRu Core‐Shell Electrocatalyst for CO‐Resilient Methanol Oxidation
Source: Adv Sci (Weinh). 2026 May 15;13(47):e75638. doi: 10.1002/advs.75638 (PMC13335856; doi:10.1002/advs.75638)
Supplement: Supplementary file 1 [file ADVS-13-e75638-s001.docx]

Supporting Information

**Trace Ru-doped PtCuRu@PtRu Core-shell Electrocatalyst for CO-resilient Methanol Oxidation**

*Tianrui Xue,^1†^ Shiyue Xing,^1†^ Zhongliang Liu,^1^ Yiting Song,^1^ Jianyi Zhang,^1^ Miaomiao Liu,^3,^* Huihui Li,^1,^* Chunzhong Li^1,2,^**

**Figure S1.** TEM images of all synthesized as-prepared samples: (a) as-prepared PtCu, (b) as-prepared PtCuRu-0.025, (c) as-prepared PtCuRu-0.05, and d) as-prepared PtCuRu-0.1.

**Figure S2.** (a, c) Atomic-resolution HAADF-STEM image of as-prepared PtCuRu-0.05. (b, d) Intensity line profiles from the atomic-resolution HAADF-STEM image (a) and (c), respectively.

**Figure S3.** (a) Cu 2*p* and (b) Ru 3*p* XPS spectra of as-prepared PtCu and as-prepared PtCuRu-0.05.

**Figure S4.** CV curves of as-prepared PtCuRu-0.05 during the electrochemical dealloying process.

**Figure S5.** (a) Atomic-resolution HAADF-STEM image of PtCuRu-0.05@PtRu. (b) Intensity line profiles from the atomic-resolution HAADF-STEM image (a).

**Figure S6.** Proportion of Pt^0^ and Pt^2+^ species in the PtCuRu-0.05@PtRu and as-prepared PtCuRu-0.05 samples.

**Figure S7.** Electrochemical performance for MOR of PtCu@Pt, PtCuRu-0.025@PtRu, PtCuRu-0.05@PtRu, PtCuRu-0.1@PtRu, and commercial Pt/C: (a) CVs in 0.1 M HClO_4_, (b) mass-normalized CVs in 0.1 M HClO_4_ and 0.5 M CH_3_OH, (c) ECSA-normalized CVs, and (d) bar charts of mass activity and specific activity.

**Figure S8.** Charge transfer coefficient and TOF of PtCuRu-0.05@PtRu, PtCu@Pt and commercial Pt/C.

**Figure S9.** Electrochemical *in-situ* ATR-SEIRAS spectrum of the MOR on (a) PtCu@Pt and (b) PtCuRu-0.05@PtRu.

**Figure S10.** Five consecutive CA tests for PtCuRu-0.05@PtRu.

**Table S1.** Atomic ratios of Pt, Cu, and Ru for as-prepared PtCuRu-x with different Ru doping levels derived from ICP-OES results.

| Samples | Pt:Cu:Ru  (feeding ratios) | Pt:Cu:Ru  (experimental) |
| --- | --- | --- |
| PtCu | 1:1:0 | 44:56:0 |
| PtCuRu-0.025 | 1:1:0.025 | 46.28:53.28:0.44 |
| PtCuRu-0.05 | 1:1:0.05 | 45.13:53.63:1.24 |
| PtCuRu-0.1 | 1:1:0.1 | 44.52:53.22:2.26 |

**Table S2.** The variation of EDS elemental content of PtCuRu-0.05@PtRu catalyst after different electrochemical tests.

| Electrochemical process | Pt (at.%) | Cu (at.%) | Ru (at.%) |
| --- | --- | --- | --- |
| As-prepared | 43.2 | 53.6 | 3.2 |
| After electrochemical dealloying | 67.3 | 22.5 | 10.2 |
| After MOR | 70.6 | 18.6 | 10.8 |
| After 1h i-t | 72.5 | 22.2 | 5.3 |
| After 3h i-t | 72.4 | 21.3 | 6.3 |
| After 5h i-t | 72.4 | 21.3 | 6.3 |

**Table S3.** ECSA comparison of the samples calculated based on the hydrogen underpotential deposition (H_upd_) and oxidation of CO (CO stripping), respectively.

| Samples | ECSA_Hupd_ (m^2^ g_Pt_^-1^) | ECSA_CO_ (m^2^ g_Pt_^-1^) | ECSA_CO_/ECSA_Hupd_ |
| --- | --- | --- | --- |
| PtCuRu-0.05@PtRu | 19.44 | 30.53 | 1.57 |
| PtCu@Pt | 18.10 | 26.25 | 1.45 |
| Commercial Pt/C | 37.75 | 44.61 | 1.18 |
